# Essential pre-treatment imaging examinations in patients with endoscopically-diagnosed early gastric cancer

**DOI:** 10.1186/1472-6947-10-33

**Published:** 2010-06-09

**Authors:** Eri Horisoko, Yoshito Tsushima, Ayako Taketomi-Takahashi, Mari Tokunaga, Keigo Endo

**Affiliations:** 1 Department of Diagnostic Radiology and Nuclear Medicine, Gunma University Graduate School of Medicine, Maebashi, Gunma, Japan; 2 Department of Radiology, Isesaki Municipal Hospital, Isesaki, Gunma, Japan

## Abstract

**Background:**

There have been no reports discussing which imaging procedures are truly necessary before treatment of endoscopically-diagnosed early gastric cancer (eEGC). The aim of this pilot study was to show which imaging examinations are essential to select indicated treatment or appropriate strategy in patients with eEGC.

**Methods:**

In 140 consecutive patients (95 men, 45 women; age, 66.4 +/- 11.3 years [mean +/- standard deviation], range, 33-90) with eEGC which were diagnosed during two years, the pre-treatment results of ultrasonography (US) and contrast-enhanced computed tomography (CT) of the abdomen, barium enema (BE) and chest radiography (CR) were retrospectively reviewed. Useful findings that might affect indication or strategy were evaluated.

**Results:**

US demonstrated useful findings in 13 of 140 patients (9.3%): biliary tract stones (n = 11) and other malignant tumors (n = 2). Only one useful finding was demonstrated on CT (pancreatic intraductal papillary mucinous tumor) but not on US (0.7%; 95% confidential interval [CI], 2.1%). BE demonstrated colorectal carcinomas in six patients and polyps in 10 patients, altering treatment strategy (11.4%; 95%CI, 6.1-16.7%). Of these, only two colorectal carcinomas were detected on CT. CR showed three relevant findings (2.1%): pulmonary carcinoma (n = 1) and cardiomegaly (n = 2). Seventy-nine patients (56%) were treated surgically and 56 patients were treated by endoscopic intervention. The remaining five patients received no treatment due to various reasons.

**Conclusions:**

US, BE and CR may be essential as pre-treatment imaging examinations because they occasionally detect findings which affect treatment indication and strategy, although abdominal contrast-enhanced CT rarely provide additional information.

## Background

Gastric cancer is one of the leading causes of cancer mortality worldwide and one of the most common malignant tumors in Japan. However, in early gastric cancer (EGC), curative treatment is possible and the five-year survival rate for EGC patients has reached 90% or greater according to not only Japanese but also European series [1-5]. EGC is defined as a cancer confined to the mucosa (m) or submucosa (sm) regardless of lymph node metastasis [6,7]. Availability of cancer screening and improved awareness of the disease has led to increased detection of EGC.

In EGC, the incidence of distant metastasis at initial diagnosis is extremely low [1], but most gastroenterologists simply assume that contrast-enhanced computed tomography (CT) of the abdomen is essential as a routine pre-treatment imaging examination. However, most diagnostic radiologists feel it is rare for pre-treatment abdominal CT to demonstrate metastatic disease which alters indicated treatment or appropriate strategy. Many benign conditions which may affect surgical procedure, such as biliary tract stones or abdominal aortic aneurysms, can usually be demonstrated by abdominal ultrasonography (US).

Surprisingly, there have been no reports discussing which imaging procedures are truly necessary before treatment of EGC. The aim of this study was to define the essential pre-treatment imaging examinations in patients with endoscopically-diagnosed EGC (eEGC).

## Methods

### Subjects

In a referral hospital of Gunma University Hospital (Isesaki Municipal Hospital, Isesaki, Japan) we retrospectively reviewed patient charts in which pre-treatment diagnosis of eEGC was made between July 2004 and June 2006. The inclusive criteria were 1) diagnosis of EGC made by endoscopic inspection and endoscopic ultrasonography (EUS) performed by an experienced endoscopist, and 2) diagnosis of adenocarcinoma made on pathological specimens obtained by punch biopsy during endoscopic examination. Some eEGCs had a final diagnosis of advanced gastric cancer after surgical resection, but postoperative diagnosis was not a criterion of case selection. 140 patients (95 men and 45 women; age 66.4 +/- 11.3 years [mean +/- standard deviation], range 33-90) met these criteria.

### Imaging procedures

Contrast-enhanced abdominal CT was performed using an eight-detector row CT scanner (LightSpeed Plus-U; GE-Yokokawa, Tokyo, Japan). Followed by a non-enhanced upper abdominal CT from the diaphragmatic dome to the lower edge of the kidney, a contrast-enhanced CT was performed from the diaphragmatic dome to the anus using nonionic contrast material (80 ml of iopamidol 300 mgI/ml in patients with 48 kg of body weight or less; 100 ml of iopamidol 300 mgI/ml in patients 48-60 kg; 100 ml of iopamidol 370 mgI/ml in patients 60-74 kg; 150 ml of iohexiol 300 mgI/ml in patients 74 kg or greater). Contrast material was intravenously administered by bolus injection using a power injector and scanning was started 100 seconds after the initiation of contrast material injection. The scan parameters for CT were as follows: section thickness, 1.25 mm; pitch 1.35; reconstruction interval, 7.5 mm; tube rotation time, 0.5 sec.

Abdominal US examination was performed after overnight fasting by an experienced sonographer (five or more years of experience) using commercially available scanners (SSD-2000, ALOKA, Tokyo, Japan) with standard transducers (3.5 MHz curvilinear array prove). All abdominal organs were carefully scanned, and any abnormal findings were recorded on hard copies.

Barium enema (BE) examination was performed using standard procedure. Chest radiograph (CR) was obtained on a conventional supine PA view. All CT, US, BE and CR images were interpreted by one of two board-certified diagnostic radiologists (EH or MT), and final imaging diagnoses were reported. The order in which CT, US, BE and CR were performed was random, and each study was independently interpreted.

### Data review

One of the authors (EH) reviewed all patient charts, including results of CT, US, CR, BE, blood cell counts and laboratory data. For purposes of this study, we noted findings which would change the treatment indication or strategy, i.e. 1) distant metastasis, particularly liver metastasis, 2) malignant tumors other than gastric cancer, and 3) any other findings which might affect indicated treatment or appropriate strategy such as an abdominal aortic aneurysm and biliary tract stones. Any abnormal findings which would change indicated treatment or strategy were recorded.

Our institutional review board does not require its approval or informed patient consent for this type of retrospective study. The Declaration of Helsinki principles were followed.

### Statistical analysis

Data was expressed by mean +/- standard deviation (SD). If necessary, 95% confidential interval (CI) was calculated. Binominal distribution was employed for the 95% CI, when appropriate. Usually, *n*p and *n*(1-p) must both exceed about 5, where p is the proportion of the observed sample having the attribute of interest, and *n *is the number observed. When it was not possible to use this approximation, we calculated the upper 95% confidence bound on the basis of the binominal distribution [8].

## Results

### Treatment selection, pathological diagnosis and patient prognosis

Of 140 patients included in this study, 79 patients (56%) were treated surgically, 51 (36%) by endoscopic mucosal resection (EMR), and five (3.6%) by endoscopic microwave coagulation therapy (EMCT). The remaining five patients (3.6%) received no treatment due to patient refusal (n = 3), advanced age (n = 1) or myelodysplastic syndrome (n = 1).

Eight of 79 surgically resected cancers had a pathologic diagnosis of advanced gastric cancer (tumor invasion of the muscularis propria [mp]). Nine patients had pathologically positive N1 lymph node metastasis, and one patient had N2 lymph node metastasis. No N3 lymph node metastasis, liver metastasis, or peritoneal dissemination was observed during laparotomy. Post operative clinical stage of gastric cancers were T1(m)N0 (n = 35), T1(sm)N0 (n = 29), T1(m)N1 (n = 1), T1(sm)N1 (n = 6), T2N0 (n = 5), T2N1 (n = 2) and T2N2 (n = 1). One of the surgical cases included a post EMR case whose pathological diagnosis was m, but had suspected to have perforation during the EMR procedure. All 51 gastric cancers treated by only EMR were pathologically proved mucosal cancers, and no additional gastrectomies were performed. EMCT (n = 5) is a tumor destructive therapy which does not allow pathological confirmation of the depth of tumor invasion. However, there were no cases of local recurrence during follow up of one year or longer.

For the operated cases, final histopathological diagnosis were well differentiated tubular adenocarcinoma (n = 32), moderately differentiated tubular adenocarcinoma (n = 16), poorly differentiated adenocarcinoma (n = 14) and signet ring cell carcinoma (n = 17). The T1(m)N1 lesion was signet ring cell carcinoma. For the EMR cases, final histopathological diagnosis were well differentiated tubular adenocarcinoma (n = 45) and moderately differentiated tubular adenocarcinoma (n = 6).

All 135 patients who received treatment were followed on an outpatient basis (follow-up period 7-36 months: 22.8 +/- 6.64). Three patients died during this period due to bone metastasis (n = 1) of gastric cancer, advanced colon cancer (n = 1) or hepatocellular carcinoma (n = 1). The other 132 patients were alive without any evidence of recurrent disease.

### Imaging findings and changes in treatment indication and strategy

The imaging examinations performed before treatment are summarized in Table 1. Abdominal contrast-enhanced CT was performed in 92% of cases, and US was performed on over half of the patients. However, nine patients (6.4%) underwent neither CT nor US.

**Table 1:** Imaging examinations before treatment (n = 140)

Examinations	No. of cases performed
CT	129 (92%)
US	74 (53%)
BE	75 (54%)
CR	140 (100%)

In nine patients (6.4%), in whom endoscopic treatments were selected, neither CT nor US were performed.

Abnormal findings demonstrated on CT or US which would potentially affect treatment indication or strategy are shown on Table 2. Biliary stones were identified in 14 patients (eight on CT and 11 on US). Three patients who had biliary stones underwent CT but not US. In all 14 patients, gastrectomy with simultaneous cholecystectomy was the chosen form of treatment. Other CT findings which would potentially affect treatment indication or strategy were one hepatocellular carcinoma and one renal cell carcinoma, but these two findings were also well demonstrated on US. A pancreatic tumor (intraductal papillary mucinous tumor) was detected by CT but not by US, even though both were performed. This is the only case in which CT demonstrated a potentially strategy-altering lesion which was not identified by US (0.7%; 95% CI, 2.1%).

**Table 2:** Abnormal findings demonstrated by imaging examinations, which may affect treatment indication or strategy for eEGC

Abnormal findings	No. of patients	Modality			
		
		CT	US	BE	CR
Biliary tract stone *a)	14	8	11	-	-
Hepatocellular carcinoma *c)	1	1	1	-	-
Renal cell carcinoma *c)	1	1	1	-	-
Pancreatic IPMT *b) *c)	1	1	0	-	-
Colorectal carcinoma *c)	6	2	0	6	-
Colorectal polyp (benign) *c)	10	0	0	10	-
Pulmonary adenocarcinoma *c)	1	-	-	-	1
Cardiomegaly	2	-	-	-	2

a) In 11 of 14 patients gallstones were confirmed on US. In the other three patients gallstones were confirmed by CT, but no abdominal US were performed. CT did not demonstrated gallstones in three patients, in whom gallstones were confirmed by US.

b) Intraductal papillary mucinous tumor.

c) All were pathologically proven.

Some CT and US findings did not change treatment or strategy: CT found suspicion of lymphadenopathy (lymph node with a maximum diameter of 1 cm or greater) [9], in the upper abdomen (N1) in eight patients. Abdominal US failed to depict all of these lesions. These lesions were not considered to be strategy-altering because none of the cases matched the criteria for EMR or EMCT on endoscopic examination, and they all underwent gastrectomy. Of theses, only one patient was pathologically confirmed to be N1. In fact, 10 of 79 surgically treated patients had pathologically positive lymph nodes in the upper abdomen, but the above described patient was the only case in which lymph node metastases was accurately diagnosed preoperatively. In seven patients, cavernous hemangiomas of the liver were suspected on US. In all seven, the diagnosis was confirmed by contrast-enhanced CT. These lesions were not considered to be strategy-altering.

BE demonstrated colorectal carcinomas in six patients and polyps in 10 patients (Table 2). Of the colorectal cancers, only two were demonstrated on CT. CT detected none of the colon polyps identified by BE. These lesions of the colon (16 of 140 patients, 11.4%: 95% CI, 6.1-16.7%) altered treatment strategy in that surgical resection of the colorectal cancers were performed simultaneously to gastrectomy, and colonic polyps were resected endoscopically.

CR findings altered treatment in three patients. Cardiomegaly was identified in two patients, and additional pre-treatment examinations were performed, finding cardiac disease which contraindicated surgical gastrectomy. Endoscopic findings of these patients fortunately met EMR criteria. CR also identified one primary adenocarcinoma of the lung, which was confirmed by chest CT and resected after gastrectomy. No lung metastasis was detected by CR.

In one case (0.71%), diffuse bone metastasis was diagnosed by bone scintigraphy. This study was performed because of markedly elevated cerium alkaline phosphatase (ALP) level. Both CT and CR failed to make this diagnosis.

## Discussion

In our series, abdominal US and BE occasionally provided useful information which may change the treatment indication or strategy for eEGC, thus they may be considered as routine pre-treatment imaging examinations. However, it was rare for useful information which would alter indicated treatment or strategy to be provided by contrast-enhanced abdominal CT.

Lymph node metastasis is the most important prognostic factor for patients with EGC [1-3,10], but once the endoscopic diagnosis of EGC is made, the presence of swollen lymph nodes in the upper abdomen does not affect indication. Indication for endoscopic therapies such as EMR and EMCT is determined by tumor histology, depth and size (mucosal cancer less than 2 cm without ulceration). Since it has been reported that lymph node metastasis is extremely rare in such small mucosal cancers [2,5,10-12], clinicians will not entertain the possibility of lymphadenopathy. If the EGC does not meet the above criteria for endoscopic treatment, gastrectomy with D2 lymph node dissection is performed, and indication for modified gastretomy is determined by intraoperative findings [5]. Added to the fact that N3 metastasis of EGC is extremely rare [10-12], detailed preoperative evaluation of lymph nodes is not essential.

Distant metastasis of EGC is extremely rare, as is synchronous liver metastasis [1]. However, since the liver is the most common site of gastric cancer metastasis [13], evaluation of the liver is necessary. In our series, however, no liver metastasis was depicted in any patients with eEGC on either CT or US as predicted. Radiologic studies may also have a potential role in detecting unrelated abnormalities which may affect indicated treatment or strategy. Some abdominal findings such as malignant abdominal tumors and biliary tract stones may be important because it opens the possibility of synchronous of surgical intervention. Fourteen (10%) of 140 patients had biliary stones. There were patients in our series who had biliary stones detected on CT and did not undergo US. However, It is not unrealistic to speculate US would have been sufficient to detect these stones.

Nine patients had other synchronous malignant tumors (6.4%) and six of them (4.3%) were colorectal carcinoma. According to a follow-up study of 1475 patients of EGC [1], 48 (3.3%) patients died due to other malignant diseases (18-186 month observation period), and the most common malignancy was colorectal carcinoma (0.7%; n = 11), so we found the high incidence of colorectal carcinoma in our series surprising [14].

To the best of our knowledge, it is not known whether synchronous surgical intervention of these diseases may provide better prognosis or quality of life, but we suspect avoiding re-laparotomy is appropriate in most cases, due to increased difficulty of the procedure and potential complications. Our results suggested that abdominal US and BE may be essential as pre-treatment imaging examinations for patients with eEGC. CR may also be necessary, since it can reveal serious conditions which may affect treatment indication or strategy, such as cardiopulmonary disease as well as lung cancer. Radiation exposure and cost of CR are low, so we can recommend its use as a routine pre-treatment examination. The cost of abdominal CT depends on the country, but it is more expensive than CR, and, according to the literature, the effective radiation dose of abdominal CT is around 20mSv [15,16], which is, again, much greater than that of CR. Omission of contrast-enhanced abdominal CT in patients with EGC would have the benefit of reduced cost and radiation exposure.

In one patient, diffuse bone metastasis was present at the initial diagnosis. We believe this case was extremely exceptional, since only 14 cases of EGC with synchronous bone metastasis have been reported in English literature [17]. According to the literature, elevated ALP can be an effective marker for bone metastasis, as it was in this case [17].

This study has several limitations: First, the number of subjects included in this study was small. In addition, since this was a retrospective study, not all examinations were performed in all patients. It is too early to conclude that pre-treatment contrast-enhanced abdominal CT can be omitted for the patients with eEGC, and prospective studies with a larger number of patients are clearly required to confirm our results. Secondly, BE was used to screen for colorectal carcinoma in this study, as was the practice in our institution during the study period. Current practice may favor colonoscopy as a screening examination. Thirdly, we did not evaluate the usefulness of US or CT in depicting ovarian metastasis from eEGC. However, only seven cases of EGC with a Kruckenberg tumor have been reported in the literature [18,19]. Finally, there were eight cases in which the postoperative diagnoses were advance gastric cancer (mp invasion). However, it has been reported that surgery with D2 lymph node dissection for mp gastric cancer can provide a cure rate similar to that for EGC [20], and well-trained endoscopists can accurately distinguish EGC from more invasive tumors by routine endoscopy alone in 90% of patients [21-23].

## Conclusions

For the patients with eEGC, abdominal US, BE and CR may be essential as pre-treatment imaging examinations because they occasionally detect findings which affect treatment indication and strategy. It was rare for contrast-enhanced abdominal CT to provide additional information. And it may be possible to eliminate contrast-enhanced abdominal CT as a preoperative examination, at least for patients receiving EMR. For gastric cancers with suspected sm invasion, the possibility of lymph node metastasis is high enough to warrant further investigation regarding the value of preoperative contrast-enhanced abdominal CT before declaring it entirely unnecessary.

## Abbreviations

eEGC: endoscopically-diagnosed early gastric cancer; US: ultrasonography; CT: computed tomography; BE: barium enema; CR: chest radiography; CI: confidential interval; EGC: early gastric cancer; m: mucosa; sm: submucosa; EUS: endoscopic ultrasonography; SD: standard deviation; EMR: endoscopic mucosal resection; EMCT: endoscopic microwave coagulation therapy; mp: muscularis propria; ALP: alkaline phosphatase.

## Competing interests

The authors declare that they have no competing interests.

## Authors' contributions

EH carried out data acquisition and analysis of this study, participated in the sequence alignment and performed manuscript preparation. YT conceived of the project and participated in the design, coordination and performed the statistical analysis. ATT participated in the design of the study and performed manuscript preparation. MT participated in the sequence of the study. KE participated in the sequence of the study. All authors read and approved the final manuscript.

## Pre-publication history

The pre-publication history for this paper can be accessed here:

http://www.biomedcentral.com/1472-6947/10/33/prepub
